# Admissions for Bronchiolitis at Children’s Hospitals Before and During the COVID-19 Pandemic

**DOI:** 10.1001/jamanetworkopen.2023.39884

**Published:** 2023-10-26

**Authors:** Kailey A. Remien, Justin Z. Amarin, Christopher M. Horvat, Ryan A. Nofziger, Christopher K. Page-Goertz, James B. Besunder, Brittany K. Potts, Michael L. Forbes, Natasha Halasa, Jonathan H. Pelletier

**Affiliations:** 1 Department of Medical Education, Akron Children’s Hospital, Akron, Ohio; 2 Division of Pediatric Infectious Diseases, Department of Pediatrics, Vanderbilt University Medical Center, Nashville, Tennessee; 3 Division of Pediatric Critical Care Medicine, Department of Critical Care Medicine, UPMC Children’s Hospital of Pittsburgh, Pittsburgh, Pennsylvania; 4 Division of Critical Care Medicine, Department of Pediatrics, Akron Children’s Hospital, Akron, Ohio; 5 Department of Pediatrics, College of Medicine, Northeast Ohio Medical University, Rootstown, Ohio; 6 Division of Hospital Medicine, Department of Pediatrics, Akron Children’s Hospital, Akron, Ohio; 7 Rebecca D. Considine Research Institute, Akron Children’s Hospital, Akron, Ohio

## Abstract

**Importance:**

The COVID-19 pandemic has been associated with a transient decrease in bronchiolitis hospitalizations compared with prepandemic patterns, but current effects remain unknown.

**Objective:**

To analyze changes in patterns of bronchiolitis admissions at US children’s hospitals during the 2020-2023 bronchiolitis seasons compared with the 2010-2019 seasons.

**Design, Setting, and Participants:**

This retrospective cross-sectional study used data from 41 US children’s hospitals in the Pediatric Health Information System database. Bronchiolitis has winter-predominant seasonality, so hospitalizations were grouped according to bronchiolitis season (from July through June). This study included all patients aged younger than 2 years admitted with a diagnosis of bronchiolitis between July 1, 2010, and June 30, 2023. Bronchiolitis seasons from July through June between 2010-2011 and 2019-2020 were classified as the prepandemic era, and seasons between 2020-2021 and 2022-2023 were classified as the pandemic era. Data analysis was performed from July 1, 2010, through June 30, 2023.

**Exposures:**

Admission date.

**Main Outcomes and Measures:**

The primary outcome was number of hospitalizations for bronchiolitis by season and month. Monthly admission counts from the prepandemic era were transformed into time series and used to train seasonal ensemble forecasting models. Forecasts were compared to monthly admissions during the pandemic era.

**Results:**

In this study, there were 400 801 bronchiolitis admissions among 349 609 patients between July 1, 2010, and June 30, 2023. The median patient age was 6 (IQR, 2-12) months; 58.7% were boys and 43.7% were White. Hospitalizations increased gradually during the prepandemic era (median, 29 309 [IQR, 26 196-34 157]), decreased 69.2% (n = 9030) in the 2020-2021 season, and increased 75.3% (n = 51 397) in the 2022-2023 season. Patients in the pandemic era were older than those in the prepandemic era (median, 7 [IQR, 3-14] vs 6 [2-12] months; *P* < .001). Intensive care unit (ICU) admissions increased from 32.2% (96 245 of 298 535) in the prepandemic era to 36.7% (37 516 of 102 266) in the pandemic era (*P* < .001). The seasonality of bronchiolitis admissions changed during the pandemic era. Admissions peaked in August 2021 (actual 5036 vs 943 [95% CI, 0-2491] forecasted) and November 2022 (actual 10 120 vs 5268 [95% CI, 3425-7419] forecasted). These findings were unchanged in sensitivity analyses excluding children with complex chronic conditions and excluding repeat admissions. In a sensitivity analysis including all viral lower respiratory tract infections in children aged younger than 5 years, there were 66 767 admissions in 2022-2023 vs 35 623 (31 301-41 002) in the prepandemic era, with the largest increase in children aged 24 to 59 months.

**Conclusions and Relevance:**

The findings of this cross-sectional study suggest that bronchiolitis hospitalizations decreased transiently and then increased markedly during the COVID-19 pandemic era. Patients admitted during the pandemic era were older and were more likely to be admitted to an ICU. These findings suggest that bronchiolitis seasonality has not yet returned to prepandemic patterns, and US hospitals should prepare for the possibility of atypical timing again in 2023.

## Key Points

**Question:**

How have hospital admissions for bronchiolitis changed since the COVID-19 pandemic?

**Findings:**

In this cross-sectional study of 41 large US children’s hospitals, there were 75% more inpatient bronchiolitis hospitalizations during the 2022-2023 bronchiolitis season compared with median bronchiolitis hospitalizations during the 2010-2019 seasons. Children admitted during the 2022-2023 season were older, more likely to be admitted to the intensive care unit, and more likely to receive noninvasive ventilation; peak admissions occurred in November 2022.

**Meaning:**

These findings suggest that bronchiolitis admissions have not yet returned to prepandemic patterns, and US hospitals should prepare for the possibility of atypical timing again in 2023.

## Teaser

This cross-sectional study examines changes in admission patterns at children’s hospitals for bronchiolitis seasons before vs during the COVID-19 pandemic.

## Introduction

Bronchiolitis is a lower respiratory tract viral infection in children aged younger than 2 years that manifests as dyspnea, cough, wheezing, and poor feeding.^1^ Bronchiolitis is of enormous public health importance across a wide spectrum of clinical severity. Worldwide, bronchiolitis has been estimated to cause as many as 3.2 million pediatric hospitalizations and 59 000 deaths annually.^2^ In the US, bronchiolitis accounts for 2.1 million annual outpatient visits,^3,4^ 18% of all pediatric hospitalizations,^5^ and 10% of pediatric intensive care unit (ICU) admissions.^5,6^ Approximately 20% of patients hospitalized with bronchiolitis are admitted to an ICU.^6,7,8^ This proportion doubled between 2010 and 2019, markedly outpacing pediatric ICU expansion.^6,7,8^ This was associated with a concomitant 7-fold increase in noninvasive ventilation (NIV) and a 13-fold increase in the use of high-flow nasal cannula (HFNC) therapy, resulting in a 63% increase in annual cost.^5,6,9,10^

Many bronchiolitis hospitalizations are attributed to respiratory syncytial virus (RSV) infection,^11^ but bronchiolitis is also caused by numerous other pathogens, including rhinovirus, influenza, and human metapneumovirus.^12,13^ These viruses are predominantly transmitted via respiratory droplets and fomites.^14,15,16^ These vectors transmit more efficiently in cool, dry air, which may contribute to the seasonality of bronchiolitis.^17,18,19,20,21^ Bronchiolitis has historically followed a winter-predominant seasonal pattern, peaking in December to February in the Northern Hemisphere^4,6,9,22^ and in May to July in the Southern Hemisphere.^23,24,25^

Early in the COVID-19 pandemic, nonpharmaceutical interventions included social distancing measures such as stay-at-home orders,^26^ school closures,^27^ and mask wearing.^28,29^ Face mask use by symptomatic individuals has been shown to reduce droplet transmission,^30^ and the use of masks and goggles or face shields by health care providers reduces nosocomial RSV infections.^31,32,33,34^ This combination of nonpharmaceutical intervention measures was associated with profound reductions in bronchiolitis admissions early in the pandemic.^35^ Whether these effects would be sustained was unclear because bronchiolitis has winter predominance even in countries such as Japan^36^ and China,^37,38^ where face masks are commonly worn.^39^ Additionally, temporary masking in the US raised the possibility of an increase in the susceptible population of infants and young children after cessation of social distancing.^28,29^ We thus sought to analyze changes in bronchiolitis admissions during the COVID-19 pandemic era (2020-2023) compared with the prepandemic era (2010-2019). We hypothesized that admissions would be higher than expected, that there would be unusual seasonality, and that patients would be older than those in previous years due to waning herd immunity from lack of exposure.

## Methods

### Study Design and Participants

This retrospective cross-sectional study used data from the Pediatric Health Information System (PHIS). The PHIS is an anonymous, quality-controlled administrative database of US children’s hospitals representing more than 20 states and many large metropolitan centers.^40^ The PHIS has been used extensively for epidemiology and quality improvement studies.^40^ This study included all admissions with an *International Classification of Diseases, Ninth Revision* (*ICD-9*) or *International Statistical Classification of Diseases and Related Health Problems, Tenth Revision* (*ICD-10*) code for bronchiolitis (466 [*ICD-9*] or J21 [*ICD-10*]) for children aged younger than 2 years admitted between July 1, 2010, and June 30, 2023. Hospitals were excluded if they did not contribute data for every bronchiolitis season between 2010-2011 and 2022-2023. There were no other exclusion criteria. This study was approved by the local institutional review board and the Children’s Hospital Association. Informed consent was waived because deidentified data were used. The study followed the Strengthening the Reporting of Observational Studies in Epidemiology (STROBE) reporting guideline.

### Data Extraction

Encounter-level data were extracted from the PHIS (including age, sex, database-reported race and ethnicity, diagnostic codes, insurance status, income, Child Opportunity Index,^41^ complex chronic conditions,^42^ length of stay, mortality, charges, and cost). Race and ethnicity data were included because the COVID-19 pandemic has previously been shown to exacerbate racial and ethnic disparities in access to care.^43^ These data were reported as Asian or Pacific Islander, Black, Hispanic, White, or other race or ethnicity (the latter of which is an option provided in the PHIS database). Invasive mechanical ventilation (IMV) and NIV were ascertained based on diagnostic codes consistent with prior work.^5,6^

### Statistical Analysis

Because bronchiolitis has winter-predominant seasonality,^4,6,9,22^ hospitalizations were grouped according to bronchiolitis season (from July through June) rather than calendar years (eg, the 2010-2011 season ranges from July 1, 2010, through June 30, 2011). Before the COVID-19 pandemic, less than 15% of annual bronchiolitis cases occurred between April and June.^35^ Because school closures in the US did not occur until the end of March 2020,^44^ the 2010-2011 through 2019-2020 bronchiolitis seasons were classified as the prepandemic era and the 2020-2021 through 2022-2023 seasons were classified as the pandemic era.

Demographics, clinical characteristics, and severity measures were described using medians and IQRs for continuous data or percentages for categorical data. Changes between seasons were tested with the Kruskal-Wallis rank sum test for continuous variables and the Pearson χ^2^ test for categorical variables.

Because there are not currently reliable methods for identifying patients receiving HFNC therapy in administrative database studies,^6^ severity of illness was grouped according to ward admission (without ICU admission), ICU admission (without NIV or IMV), NIV (without IMV), and IMV. Thus, the ICU admission group included children receiving HFNC therapy and children admitted to the ICU for other reasons. Intensive care unit admissions included pediatric and neonatal ICUs. Cost analyses were abstracted from hospital charges, determined by the cost-to-charge ratio submitted to the US Centers for Medicare & Medicaid Services (CMS) annually, and adjusted by the CMS wage index or price index according to hospital zip code in the PHIS.^6,40^ Costs were adjusted for the annual US gross domestic product (GDP) provided by the Bureau of Economic Analysis^45^ and expressed in 2023 dollars.^46^

The primary outcome of interest was number of hospitalizations for bronchiolitis by season and month during the COVID-19 pandemic. Therefore, monthly bronchiolitis admissions across the PHIS database were analyzed as a time series. Admissions from the prepandemic era were used to train ensemble forecasting models using autoregressive integrated moving average^47^ and neural network^48^ algorithms. These models assume that seasonality follows a consistent yearly cycle that can be determined by autocorrelation within the training data.^49^ Models were weighted on time series cross-validation,^49^ similar to prior work.^35^ This approach weights models based on the error of rolling forecasts through the training data, and it requires several complete seasonal cycles for optimal performance.^49^ Training forecasting models on the prepandemic era allows a hypothetical counterfactual analysis to illustrate changes in seasonality that occurred during the pandemic.^35^ Actual pandemic era admission counts were compared against model forecasts and 95% CIs.

We conducted 3 sensitivity analyses. First, to determine whether changes in admission rates were due to loss of herd immunity in well children or increased susceptibility of medically fragile children, we excluded patients with complex chronic conditions. Second, because some prior studies have defined bronchiolitis as the first episode of symptoms,^50,51^ we included only the first admission per patient. Third, because older patients can be affected by the same viruses that cause infantile bronchiolitis,^2,3,52^ we conducted an analysis including all children aged younger than 5 years with a diagnosis of bronchiolitis or viral pneumonia (466 or 480 [*ICD-9*] and J12 or J21 [*ICD-10*]) to better understand the association between viral lower respiratory tract infections and changes in admissions during the COVID-19 pandemic.

All statistical analyses were conducted in R, version 4.3.1 (R Project for Statistical Computing). An α value of .05 was used for statistical significance. Data analysis was performed from July 1, 2010, through June 30, 2023.

## Results

### Incidence

**Figure 1.**
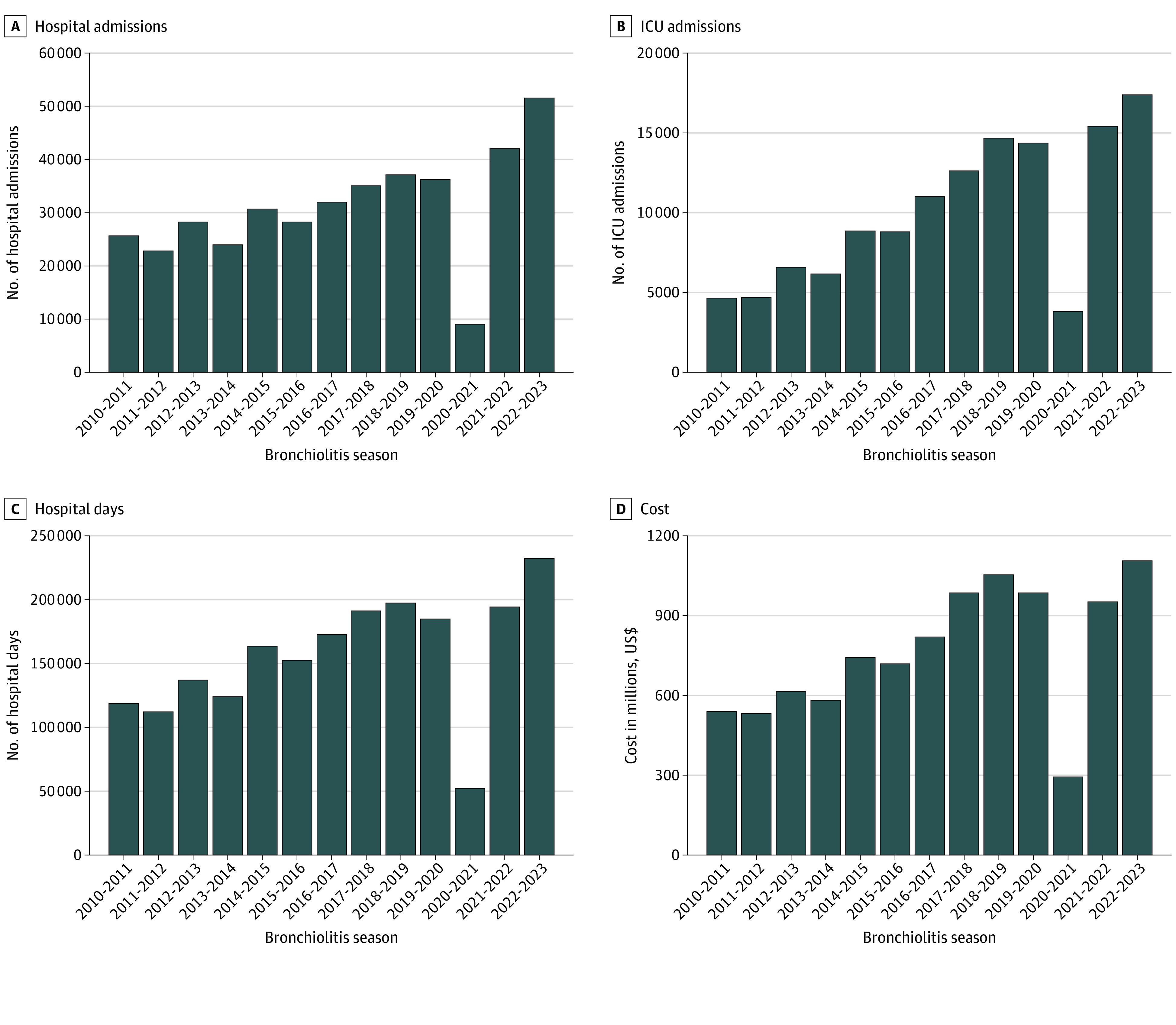
Hospital Admissions, Bed Utilization, and Cost by Bronchiolitis Season A to D, Number of hospital admissions (A), intensive care unit (ICU) admissions (B), hospital days (C), and inflation-adjusted cost (D). The bar height represents the value for each panel. In the x-axis for each facet, bronchiolitis season is considered July through June of the first and second year, respectively, of each year range. Each facet has a different y-axis scale to show trends.

There were 400 801 inpatient admissions for bronchiolitis among 349 609 patients across 41 hospitals between July 1, 2010, and June 30, 2023. The median patient age was 6 (IQR, 2-12) months; 41.3% were girls and 58.7% were boys. In addition, 3.4% of patients were Asian or Pacific Islander, 19.3% were Black, 24.7% were Hispanic, 43.7% were White, and 8.9% were of other race or ethnicity. Demographic data are presented in the Table. The median number of annual admissions for bronchiolitis was 29 309 (IQR, 26 196-34 157) in the prepandemic era between the 2010-2011 and 2019-2020 bronchiolitis seasons (Figure 1). There was a 69.2% reduction in admissions (n = 9030) during the 2020-2021 season. However, admissions increased during the 2021-2022 and 2022-2023 seasons, with a 75.3% increase (n = 51 397) above the prepandemic median in 2022-2023. Of the 41 hospitals, 39 had more admissions in the 2022-2023 season than their average from the prepandemic era (median increase, 83.9% [IQR, 33.7%-120.7%]). Hospital and ICU days followed similar patterns and were 47.1% and 45.2% higher, respectively, in the 2022-2023 season compared with the prepandemic median (Figure 1). Inflation-adjusted hospitalization costs during the 2022-2023 season were $375.6 million higher than the prepandemic median (Figure 1).

**Table.** Patient Characteristics

Characteristic	No. (%) of bronchiolitis admissions^a^			*P* value
	Overall (N = 400 801)	Prepandemic (n = 298 535)	Pandemic (n = 102 266)	
Age, median (IQR), mo	6 (2-12)	6 (2-12)	7 (3-14)	<.001^b^
Age group, mo				
0-3	143 698 (35.9)	111 851 (37.5)	31 847 (31.1)	<.001^c^
4-11	146 147 (36.5)	109 882 (36.8)	36 265 (35.5)	
12-23	110 956 (27.7)	76 802 (25.7)	34 154 (33.4)	
Race and ethnicity				
Asian or Pacific Islander	13 749 (3.4)	10 137 (3.4)	3612 (3.5)	<.001^c^
Black	77 332 (19.3)	59 211 (19.8)	18 121 (17.7)	
Hispanic	98 960 (24.7)	73 034 (24.5)	25 926 (25.4)	
White	175 033 (43.7)	128 123 (42.9)	46 910 (45.9)	
Other^d^	35 727 (8.9)	28 030 (9.4)	7697 (7.5)	
Sex				
Female	165 530 (41.3)	123 772 (41.5)	41 758 (40.8)	<.001^c^
Male	235 237 (58.7)	174 741 (58.5)	60 496 (59.2)	
Unknown, No.	34	22	12	
Complex chronic condition	84 588 (21.1)	64 003 (21.4)	20 585 (20.1)	<.001^c^
Child Opportunity Index score				
Median (IQR)	43 (19-71)	42 (18-69)	47 (22-74)	<.001^b^
Unknown, No.	1134	979	155	
Length of stay, d				
Median (IQR)	3 (2-5)	3 (2-5)	3 (2-4)	<.001^b^
Unknown, No.	1	1	0	
Cost, $US				
Median (IQR)	9524 (5486-18 260)	9439 (5422-18 462)	9760 (5684-17 725)	<.001^b^
Unknown, No.	18	18	0	
Acuity level				
Ward	267 040 (66.6)	202 290 (67.8)	64 750 (63.3)	<.001^c^
ICU (without NIV or IMV)	78 069 (19.5)	55 496 (18.6)	22 573 (22.1)	
NIV (without IMV)	29 943 (7.5)	20 371 (6.8)	9572 (9.4)	
IMV	25 749 (6.4)	20 378 (6.8)	5371 (5.3)	
In-hospital mortality	812 (0.20)	643 (0.22)	169 (0.17)	.002^c^

Abbreviations: ICU, intensive care unit; IMV, invasive mechanical ventilation; NIV, noninvasive ventilation.

^a^ The prepandemic era ranged from July 1, 2010, to June 30, 2020; the pandemic era ranged from July 1, 2010, to June 30, 2023.

^b^ Wilcoxon rank sum test.

^c^ Pearson χ^2^ test.

^d^ Includes patients who selected the category “Other” in the Pediatric Health Information System database as well as those who declined to provide race and ethnicity information.

### Demographics

**Figure 2.**
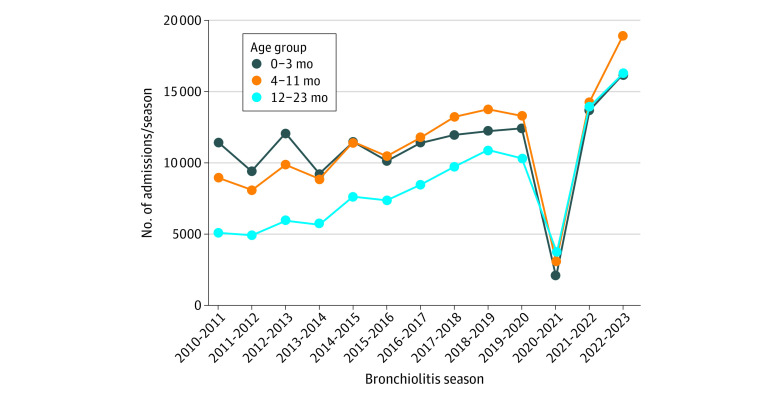
Annual Bronchiolitis Admissions by Age Group In the x-axis, bronchiolitis season is considered July through June of the first and second year, respectively, of each year range.

Briefly, compared with the prepandemic era, children admitted in the pandemic era were older (median, 7 [IQR, 3-14] vs 6 [2-12] months; *P* < .001; Table). Although there was an increase in admissions among all age groups, the difference was most notable among children aged 12 to 23 months, in whom admissions increased 2.2-fold in the 2022-2023 season compared with the prepandemic era (Figure 2). Compared with the prepandemic era, there were fewer admissions for children with complex chronic conditions and more admissions for children of White race or Hispanic ethnicity (Table). During the pandemic era, length of stay was slightly shorter than in the prepandemic era (Table), but per-admission inflation-adjusted costs were higher (median, $9760 [IQR, $5684-$17 725] vs $9439 [$5422-$18 462]; *P* < .001; Table).

### Severity of Illness

**Figure 3.**
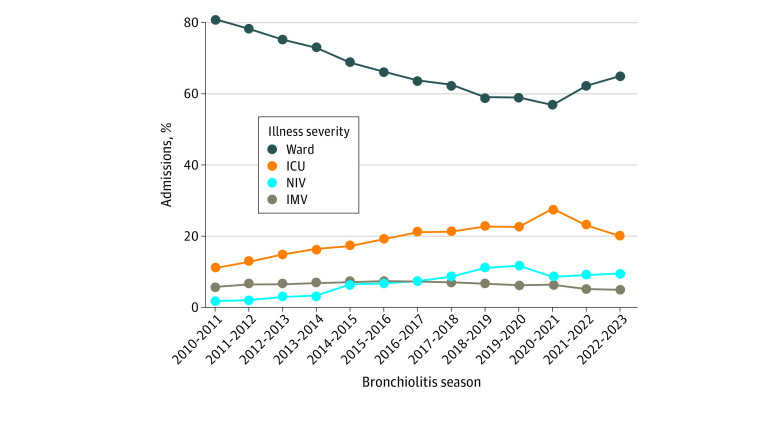
Severity of Illness by Season In the x-axis, bronchiolitis season is considered July through June of the first and second year, respectively, of each year range. ICU indicates intensive care unit; IMV, invasive mechanical ventilation; NIV, noninvasive ventilation.

There was an annual decrease in the percentage of hospitalizations admitted to the pediatric ward during the prepandemic era (81.2% in 2010 vs 59.1% in 2019; *P* < .001 by Pearson χ^2^ test; Figure 3). This was accompanied by an increase in patients admitted to the ICU without NIV or IMV (11.1% in 2010 vs 22.7% in 2019; *P* < .001) and in those receiving NIV (1.8% in 2010 vs 11.8% in 2019; *P* < .001) (Figure 3). There was a smaller increase in those receiving IMV (5.9% in 2010 vs 6.3% in 2019; *P* < .001; Figure 3). However, these trends reversed during the pandemic era, with increased admissions to the pediatric ward (65.2% in 2022 vs 59.1% in 2019; *P* < .001) and concomitant decreases in ICU admissions, NIV, and IMV (Figure 3). This reversal was not explained by differences in patient age during the pandemic era, as similar trends were seen across all age groups (eFigure 1 in Supplement 1).

### Seasonality

**Figure 4.**
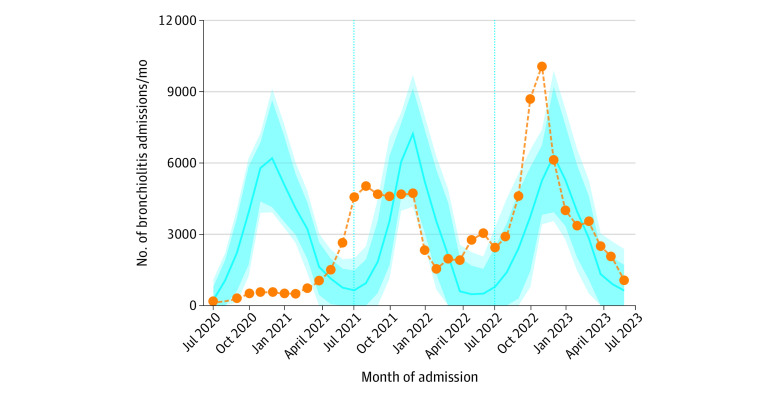
Seasonality of Bronchiolitis Admissions Between the 2020-2021 and 2022-2023 Seasons The blue line represents the forecasted monthly admission count. The dotted orange line indicates the actual number of monthly admissions. The dark blue and light blue shaded regions represent the model 80% and 95% CIs, respectively. Model weighting was performed with an optimized autoregessive integrated moving average of 51.0% and a neural network of 49.0%. Vertical lines separate the bronchiolitis seasons.

Forecasting model fitting data are shown in eFigure 2 in Supplement 1. The prepandemic bronchiolitis seasons displayed winter-predominant seasonality, with admissions peaking in December through February (eFigure 2 in Supplement 1). The pandemic bronchiolitis seasons displayed atypical seasonality (Figure 4). There was a marked reduction in bronchiolitis admissions during the 2020 season, with 9030 admissions compared with the 35 418 (95% CI, 16 537-55 799) forecasted (Figure 4). This was followed by an atypical peak in admissions in August 2021, with 5036 admissions compared with the 943 (95% CI, 0-2491) forecasted (Figure 4). Monthly admissions did not display winter-predominant seasonality during the 2021 season but remained elevated until January 2022 (Figure 4). The 2022-2023 season displayed fall-predominant seasonality. Admissions peaked in November 2022, with 10 120 admissions compared with the 5268 (95% CI, 3425-7419) forecasted (Figure 4). Admissions decreased to within model 95% confidence limits beginning in December 2022 (Figure 4). Similar seasonality was seen in all age groups.

### Sensitivity Analyses

Demographics for the sensitivity analysis excluding children with complex chronic conditions are presented in eTable 1 in Supplement 1. Briefly, there were 316 213 hospital admissions among 286 454 patients. There were 41 419 admissions in the 2022-2023 season compared with 23 372 (IQR, 21 265-26 291) in the prepandemic era (eFigure 3 in Supplement 1). There were similar trends in patient age, severity of illness, and seasonality as in the main analysis.

Demographic data for the sensitivity analysis including only the first admission per patient are shown in eTable 2 in Supplement 1. Briefly, there were 349 609 hospital admissions among 349 609 patients. There were 43 945 admissions in the 2022-2023 season compared with 26 035 (IQR, 24 063-29 173) in the prepandemic era (eFigure 4 in Supplement 1). There were similar trends in patient age, severity of illness, and seasonality as in the main analysis.

The demographics for the sensitivity analysis including bronchiolitis and viral pneumonia in patients aged younger than 5 years are shown in eTable 3 in Supplement 1. Briefly, there were 492 286 hospital admissions among 417 991 patients. There were 66 767 admissions in the 2022-2023 season compared with 35 623 (IQR, 31 301-41 002) in the prepandemic era (eFigure 5 in Supplement 1). There were increased admissions for all age groups in the 2021-2022 and 2022-2023 seasons, most notably in the group aged 24 to 59 months (13 973 admissions in the 2022-2023 season vs 5169 [IQR, 4191-5795] in the prepandemic era; eFigure 6 in Supplement 1). There were similar trends in severity of illness and seasonality as in the main analysis.

## Discussion

To our knowledge, this large database analysis is the first nationwide study to examine the changes in bronchiolitis incidence and demographics in the US during the 2020-2023 seasons. We observed that the 2022-2023 season was associated with higher-than-typical admissions. We also noted an increase in the number of ICU admissions and higher use of NIV. These findings were stable in sensitivity analyses excluding children with complex chronic conditions and excluding repeat admissions. The findings were magnified in a sensitivity analysis including viral bronchiolitis and viral pneumonia in children aged younger than 5 years, with markedly higher-than-forecasted admissions in children aged 24 to 59 months. This study supports prior work suggesting that transient social distancing may result in waning population immunity, and these findings highlight the importance of sustained interventions, such as respiratory viral vaccines^53^ and monoclonal antibodies,^54^ to help reduce the burden of illness.

Increased admissions were seen in all age groups, but the greatest change was in older infants and toddlers. This analysis confirms previous single-center work showing similar demographic trends.^55^ Excess admissions in older infants do not seem to be due to more admissions for children with medical complexity; findings were similar in a sensitivity analysis excluding children with complex chronic conditions. Social distancing and masking during the pandemic may be linked to this demographic shift. Previous investigators posited that these measures would temporarily decrease viral transmission,^31,32,33,34^ result in waning immunity,^56^ and thus increase the susceptible population.^57^ Increased admissions in the sensitivity analysis including preschool children in our study support this hypothesis, as do increased RSV-associated hospital admissions in adults in the 2022-2023 season.^58^ Forecasting future bronchiolitis seasons in the post-COVID era remains challenging. However, taken together, these data suggest that the 2023-2024 bronchiolitis season may regress to typical prepandemic levels as population immunity returns to historical norms.

Bronchiolitis admissions continue to disproportionately affect ICUs.^6^ Although the ICU admission proportion decreased between the 2020-2021 and 2022-2023 seasons, the combination of increased case burden and increased care intensity more than tripled the number of pediatric ICU admissions for bronchiolitis during the 2022-2023 season compared with the 2010-2011 season. While pediatric ICUs in the US have expanded over the past decade,^59^ bronchiolitis ICU admissions are markedly outpacing ICU growth.^6^ In the present study, the rise in ICU admissions is likely mediated by increases in the use of NIV and HFNC,^6,10^ given that IMV has declined since the pandemic in all age groups. However, given the lack of a validated severity of illness score for infants with bronchiolitis outside of the ICU,^60^ it remains unclear whether these changes represent variability in severity of illness, clinician practices, or both. Without intervention, these trends are likely to result in recurrent seasonal shortages of ICU beds.^61,62^ Thus, study of ventilatory support in bronchiolitis and identification of which patients might be safely treated with HFNC therapy outside of the ICU remains an urgent research need.

Bronchiolitis admissions displayed unusual seasonality during the COVID-19 pandemic. There was approximately one-third the typical number of total admissions during the 2020-2021 season, with very few cases during the winter. Cases increased following relaxation of social distancing measures and cessation of the US Centers for Disease Control and Prevention (CDC) mask mandate in March 2021^28^ and peaked in August 2021. Bronchiolitis admissions displayed a less clear association with masking during the 2021 season, possibly due to more variable enforcement of the second CDC mask mandate between July 2021 and February 2022 combined with reopening of schools and childcare centers in most states.^26,27,28,29^ The 2022-2023 season displayed fall-predominant seasonality, with admissions peaking in November 2022. It is unclear whether this represents a sustained shift in RSV seasonality. However, hospitals should prepare for the possibility of an earlier-than-usual increase in bronchiolitis cases in fall 2023, and RSV surveillance efforts should begin in the early fall of 2023 with attention to timely administration of monoclonal antibody therapy to eligible patients.^54,63^

### Limitations

This study has some limitations. First, there is no comprehensive database of pediatric admissions in the US. This study only included data from 41 large academic children’s hospitals. Because the PHIS is not geographically comprehensive, patient relocation or changes in referral patterns during the study may affect the results. For example, if community hospitals increased the proportion of children referred to children’s hospitals during the COVID-19 pandemic, it would increase the number of admissions captured in the PHIS. Thus, although our report of an increase in bronchiolitis admissions reported in the pandemic era is important for understanding resource strain, it is not possible to determine whether it is also representative of trends at community hospitals. Second, although ICU admission and IMV are useful proxies for severity of illness, the only validated severity of illness score for bronchiolitis requires granular vital sign information not available in administrative databases.^60^ Thus, we cannot determine whether changes in admission and ventilation rates represent changes in patient acuity or clinician practices. Third, bronchiolitis admissions were identified based on *ICD-9* and *ICD-10* codes, which may underestimate the true number of bronchiolitis admissions.^64^ Fourth, while costs were abstracted from hospital charges and adjusted for inflation using recommended practices,^46^ the COVID-19 pandemic also affected the US GDP,^65^ which may affect cost estimates. Fifth, PHIS hospitals are heterogeneously distributed across the US. Bronchiolitis season historically occurs earlier in southern and eastern states^22^; however, this study cannot capture local nuances in bronchiolitis seasonality due to our data usage agreement.

## Conclusions

In this cross-sectional study, the COVID-19 pandemic was associated with a transient decrease in bronchiolitis admissions during the 2020-2021 season, followed by higher-than-usual admissions in the 2021-2022 and 2022-2023 seasons with earlier-than-usual seasonal peaks. Patients admitted in the pandemic era were older, more likely to be admitted to a pediatric ICU and receive NIV, and less likely to receive IMV compared with the prepandemic era. The seasonality of bronchiolitis admissions does not yet appear to be stable, and US hospitals should prepare for the possibility of atypical timing again in 2023.

## Notes

Supplement 1. **eTable 1.** Demographics Table for Sensitivity Analysis Excluding Children With Complex Chronic Conditions **eTable 2.** Demographics Table for Sensitivity Analysis Including Only First Admission per Patient **eTable 3.** Demographics Table for Sensitivity Analysis Including Bronchiolitis and Viral Pneumonia for Children Aged Younger Than 5 Years **eFigure 1.** Severity of Illness by Season and Age Group **eFigure 2.** Seasonality of Bronchiolitis Admissions Between 2010-2023 With Ensemble Forecast Model Fitting Data **eFigure 3.** Bronchiolitis Hospital Admissions, Bed Utilization, and Cost by Season for Sensitivity Analysis Excluding Complex Chronic Conditions **eFigure 4.** Bronchiolitis Hospital Admissions, Bed Utilization, and Cost by Season for Sensitivity Analysis Including Only First Admission per Patient **eFigure 5.** Bronchiolitis Hospital Admissions, Bed Utilization, and Cost by Season for Sensitivity Analysis Including Bronchiolitis and Viral Pneumonia for Patients Aged Younger Than 5 Years **eFigure 6.** Annual Bronchiolitis Admissions by Age Group for Sensitivity Analysis Including Bronchiolitis and Viral Pneumonia for Patients Aged Younger Than 5 Years

Supplement 2.
Data Sharing Statement
