# Supplementary material for: Admissions for Bronchiolitis at Children’s Hospitals Before and During the COVID-19 Pandemic
Source: JAMA Netw Open. 2023 Oct 26;6(10):e2339884. doi: 10.1001/jamanetworkopen.2023.39884 (PMC10603547; doi:10.1001/jamanetworkopen.2023.39884)
Supplement: Supplement 1. — eTable 1. Demographics Table for Sensitivity Analysis Excluding Children With Complex Chronic Conditions eTable 2. Demographics Table for Sensitivity Analysis Including Only First Admission per Patient eTable 3. Demographics Table for Sensitivity Analysis Including Bronchiolitis and Viral Pneumonia for Children Aged Younger Than 5 Years eFigure 1. Severity of Illness by Season and Age Group eFigure 2. Seasonality of Bronchiolitis Admissions Between 2010-2023 With Ensemble Forecast Model Fitting Data eFigure 3. Bronchiolitis Hospital Admissions, Bed Utilization, and Cost by Season for Sensitivity Analysis Excluding Complex Chronic Conditions eFigure 4. Bronchiolitis Hospital Admissions, Bed Utilization, and Cost by Season for Sensitivity Analysis Including Only First Admission per Patient eFigure 5. Bronchiolitis Hospital Admissions, Bed Utilization, and Cost by Season for Sensitivity Analysis Including Bronchiolitis and Viral Pneumonia for Patients Aged Younger Than 5 Years eFigure 6. Annual Bronchiolitis Admissions by Age Group for Sensitivity Analysis Including Bronchiolitis and Viral Pneumonia for Patients Aged Younger Than 5 Years [file jamanetwopen-e2339884-s001.pdf]

Supplementary Online Content

Remien KA, Amarin JZ, Horvat CM, et al. Admissions for bronchiolitis at children's hospitals before vs during the COVID-19 pandemic. *JAMA Netw Open*. 2023;6(10):e2339884. doi:10.1001/jamanetworkopen.2023.39884

eTable 1. Demographics Table for Sensitivity Analysis Excluding Children With Complex Chronic Conditions

eTable 2. Demographics Table for Sensitivity Analysis Including Only First Admission per Patient

eTable 3. Demographics Table for Sensitivity Analysis Including Bronchiolitis and Viral Pneumonia for Children Aged Younger Than 5 Years

eFigure 1. Severity of Illness by Season and Age Group

eFigure 2. Seasonality of Bronchiolitis Admissions Between 2010-2023 With Ensemble Forecast Model Fitting Data

eFigure 3. Bronchiolitis Hospital Admissions, Bed Utilization, and Cost by Season for Sensitivity Analysis Excluding Complex Chronic Conditions

eFigure 4. Bronchiolitis Hospital Admissions, Bed Utilization, and Cost by Season for Sensitivity Analysis Including Only First Admission per Patient

eFigure 5. Bronchiolitis Hospital Admissions, Bed Utilization, and Cost by Season for Sensitivity Analysis Including Bronchiolitis and Viral Pneumonia for Patients Aged Younger Than 5 Years

eFigure 6. Annual Bronchiolitis Admissions by Age Group for Sensitivity Analysis Including Bronchiolitis and Viral Pneumonia for Patients Aged Younger Than 5 Years

This supplementary material has been provided by the authors to give readers additional information about their work.

© 2023 Remien KA et al. *JAMA Network Open*.

eTable 1. Demographics Table for Sensitivity Analysis Excluding Children With Complex Chronic Conditions

Characteristic	Overall, N = 316,213[1]	Era: Pre-Pandemic (7/2010-6/2020), N = 234,532[1]	Era: Pandemic (7/2020-6/2023), N = 81,681[1]	p-value
**Age (months)**	6.0 (2.0, 12.0)	5.0 (2.0, 11.0)	7.0 (3.0, 14.0)	<0.001[2]
**Age Group**				<0.001[3]
0-3 Months	117,696 (37.2%)	92,277 (39.3%)	25,419 (31.1%)	
4-11 Months	112,886 (35.7%)	84,198 (35.9%)	28,688 (35.1%)	
12-23 Months	85,631 (27.1%)	58,057 (24.8%)	27,574 (33.8%)	
**Race & Ethnicity**				<0.001[3]
White	139,085 (44.0%)	101,172 (43.1%)	37,913 (46.4%)	
Black	58,554 (18.5%)	44,804 (19.1%)	13,750 (16.8%)	
Hispanic	78,806 (24.9%)	57,994 (24.7%)	20,812 (25.5%)	
AAPI	11,047 (3.5%)	8,068 (3.4%)	2,979 (3.6%)	

© 2023 Remien KA et al. *JAMA Network Open*.

Characteristic	**Overall**, N = 316,213[1]	Era<br>**Pre-Pandemic (7/2010-6/2020)**, N = 234,532[1]	 **Pandemic (7/2020-6/2023)**, N = 81,681[1]	**p-value**
Other	28,721 (9.1%)	22,494 (9.6%)	6,227 (7.6%)	
**Sex**				<0.001[3]
Female	130,287 (41.2%)	97,147 (41.4%)	33,140 (40.6%)	
Male	185,899 (58.8%)	137,366 (58.6%)	48,533 (59.4%)	
(Unknown)	27	19	8	
**Complex Chronic Condition**				
No	316,213 (100.0%)	234,532 (100.0%)	81,681 (100.0%)	
**Childhood Opportunity Index Score**	43 (19, 71)	42 (18, 70)	48 (23, 75)	<0.001[2]
(Unknown)	835	727	108	
**LOS (Days)**	3 (2, 4)	3 (2, 4)	2 (2, 4)	<0.001[2]

© 2023 Remien KA et al. *JAMA Network Open*.

Characteristic	**Overall**, N = 316,213[1]	**Era** **Pre-Pandemic (7/2010-6/2020)**, N = 234,532[1]	**Pandemic (7/2020-6/2023)**, N = 81,681[1]	p-value
**Cost ($)**	8,418 (5,033, 14,854)	8,310 (4,961, 14,855)	8,730 (5,259, 14,854)	<0.001[2]
(Unknown)	16	16	0	
**Acuity Level**				<0.001[3]
Ward	223,311 (70.6%)	168,751 (72.0%)	54,560 (66.8%)	
ICU (without NIV or IMV)	58,639 (18.5%)	41,196 (17.6%)	17,443 (21.4%)	
NIV (without IMV)	22,684 (7.2%)	15,265 (6.5%)	7,419 (9.1%)	
IMV	11,579 (3.7%)	9,320 (4.0%)	2,259 (2.8%)	
**In-Hospital Mortality**	54 (0.02%)	40 (0.02%)	14 (0.02%)	0.987[3]

[1] Median (IQR); n (%)

[2] Wilcoxon rank sum test

[3] Pearson's Chi-squared test

AAPI: Asian American and Pacific Islander
ICU: Intensive Care Unit
IMV: Invasive Mechanical Ventilation
NIV: Non-Invasive Ventilation

© 2023 Remien KA et al. *JAMA Network Open*.

eTable 2. Demographics Table for Sensitivity Analysis Including Only First Admission per Patient

		Era		
**Characteristic**	**Overall**, N = 349,609[1]	**Pre-Pandemic (7/2010-6/2020)**, N = 261,409[1]	**Pandemic (7/2020-6/2023)**, N = 88,200[1]	**p-value**
**Age (months)**	5.0 (2.0, 11.0)	5.0 (2.0, 11.0)	6.0 (2.0, 13.0)	<0.001[2]
**Age Group**				<0.001[3]
0-3 Months	137,765 (39.4%)	107,240 (41.0%)	30,525 (34.6%)	
4-11 Months	125,118 (35.8%)	94,244 (36.1%)	30,874 (35.0%)	
12-23 Months	86,726 (24.8%)	59,925 (22.9%)	26,801 (30.4%)	
**Race & Ethnicity**				<0.001[3]
White	154,265 (44.1%)	113,567 (43.4%)	40,698 (46.1%)	
Black	65,808 (18.8%)	50,491 (19.3%)	15,317 (17.4%)	
Hispanic	85,515 (24.5%)	63,387 (24.2%)	22,128 (25.1%)	
AAPI	11,788 (3.4%)	8,709 (3.3%)	3,079 (3.5%)	

© 2023 Remien KA et al. *JAMA Network Open*.

Characteristic	**Overall**, N = 349,609[1]	Era<br>**Pre-Pandemic (7/2010-6/2020)**, N = 261,409[1]	 **Pandemic (7/2020-6/2023)**, N = 88,200[1]	p-value
Other	32,233 (9.2%)	25,255 (9.7%)	6,978 (7.9%)	
**Sex**				<0.001[3]
Female	145,683 (41.7%)	109,354 (41.8%)	36,329 (41.2%)	
Male	203,894 (58.3%)	152,035 (58.2%)	51,859 (58.8%)	
(Unknown)	32	20	12	
**Complex Chronic Condition**	65,742 (18.8%)	49,684 (19.0%)	16,058 (18.2%)	<0.001[3]
**Childhood Opportunity Index Score**	43 (19, 71)	42 (19, 70)	47 (23, 74)	<0.001[2]
(Unknown)	1,020	882	138	
**LOS (Days)**	3 (2, 5)	3 (2, 5)	3 (2, 4)	<0.001[2]
**Cost ($)**	9,323 (5,387, 17,773)	9,227 (5,318, 17,931)	9,608 (5,598, 17,364)	<0.001[2]
(Unknown)	3	3	0	

© 2023 Remien KA et al. *JAMA Network Open*.

Characteristic	Overall, N = 349,609[1]	Era: Pre-Pandemic (7/2010-6/2020), N = 261,409[1]	Era: Pandemic (7/2020-6/2023), N = 88,200[1]	p-value
**Acuity Level**				<0.001[3]
Ward	234,086 (67.0%)	178,047 (68.1%)	56,039 (63.5%)	
ICU (without NIV or IMV)	67,312 (19.3%)	47,982 (18.4%)	19,330 (21.9%)	
NIV (without IMV)	25,414 (7.3%)	17,300 (6.6%)	8,114 (9.2%)	
IMV	22,797 (6.5%)	18,080 (6.9%)	4,717 (5.3%)	
**In-Hospital Mortality**	641 (0.18%)	507 (0.19%)	134 (0.15%)	0.012[3]

[1] Median (IQR); n (%)

[2] Wilcoxon rank sum test

[3] Pearson's Chi-squared test

AAPI: Asian American and Pacific Islander
ICU: Intensive Care Unit
IMV: Invasive Mechanical Ventilation
NIV: Non-Invasive Ventilation

© 2023 Remien KA et al. *JAMA Network Open*.

eTable 3. Demographics Table for Sensitivity Analysis Including Bronchiolitis and Viral Pneumonia for Children Aged Younger Than 5 Years

		Era		
**Characteristic**	**Overall**, N = 492,286[1]	**Pre-Pandemic (7/2010-6/2020)**, N = 360,004[1]	**Pandemic (7/2020-6/2023)**, N = 132,282[1]	**p-value**
**Age (months)**	8.0 (3.0, 17.0)	7.0 (2.0, 16.0)	11.0 (4.0, 20.0)	<0.001[2]
**Age Group**				<0.001[3]
0-3 Months	146,298 (29.7%)	113,862 (31.6%)	32,436 (24.5%)	
4-11 Months	150,251 (30.5%)	113,006 (31.4%)	37,245 (28.2%)	
12-23 Months	118,728 (24.1%)	82,750 (23.0%)	35,978 (27.2%)	
24-59 Months	77,009 (15.6%)	50,386 (14.0%)	26,623 (20.1%)	
**Race & Ethnicity**				<0.001[3]
White	215,308 (43.7%)	155,022 (43.1%)	60,286 (45.6%)	
Black	92,684 (18.8%)	69,709 (19.4%)	22,975 (17.4%)	
Hispanic	122,925 (25.0%)	88,867 (24.7%)	34,058 (25.7%)	

© 2023 Remien KA et al. *JAMA Network Open*.

Characteristic	**Overall**, N = 492,286[1]	Era<br>**Pre-Pandemic (7/2010-6/2020)**, N = 360,004[1]	 **Pandemic (7/2020-6/2023)**, N = 132,282[1]	p-value
AAPI	17,989 (3.7%)	12,856 (3.6%)	5,133 (3.9%)	
Other	43,380 (8.8%)	33,550 (9.3%)	9,830 (7.4%)	
**Sex**				0.004[3]
Female	207,891 (42.2%)	152,469 (42.4%)	55,422 (41.9%)	
Male	284,356 (57.8%)	207,511 (57.6%)	76,845 (58.1%)	
(Unknown)	39	24	15	
**Complex Chronic Condition**	120,382 (24.5%)	89,755 (24.9%)	30,627 (23.2%)	<0.001[3]
**Childhood Opportunity Index Score**	44 (20, 72)	43 (19, 70)	48 (23, 75)	<0.001[2]
(Unknown)	1,446	1,231	215	
**LOS (Days)**	3 (2, 5)	3 (2, 5)	3 (2, 5)	<0.001[2]
(Unknown)	1	1	0	

© 2023 Remien KA et al. *JAMA Network Open*.

Characteristic	Overall, N = 492,286[1]	Era<br>Pre-Pandemic (7/2010-6/2020), N = 360,004[1]	Pandemic (7/2020-6/2023), N = 132,282[1]	p-value
**Cost ($)**	9,838 (5,637, 19,193)	9,764 (5,569, 19,447)	10,022 (5,821, 18,557)	<0.001[2]
(Unknown)	21	21	0	
**Acuity Level**				<0.001[3]
Ward	324,592 (65.9%)	241,207 (67.0%)	83,385 (63.0%)	
ICU (without NIV or IMV)	94,952 (19.3%)	66,154 (18.4%)	28,798 (21.8%)	
NIV (without IMV)	37,722 (7.7%)	25,451 (7.1%)	12,271 (9.3%)	
IMV	35,020 (7.1%)	27,192 (7.6%)	7,828 (5.9%)	
**In-Hospital Mortality**	1,493 (0.30%)	1,155 (0.32%)	338 (0.26%)	<0.001[3]

[1] Median (IQR); n (%)

[2] Wilcoxon rank sum test

[3] Pearson's Chi-squared test

AAPI: Asian American and Pacific Islander
ICU: Intensive Care Unit
IMV: Invasive Mechanical Ventilation
NIV: Non-Invasive Ventilation

© 2023 Remien KA et al. *JAMA Network Open*.

eFigure 1. Severity of Illness by Season and Age Group

The x-axis shows the bronchiolitis seasons. The y-axis shows the percentage of admissions. The lines show the percentage of admissions grouped by illness severity. The color scale to the right corresponds to the illness severity and corresponds to both the line plot and the numbers below the line chart. Each facet shows an age group, specified at the top in the gray bar.

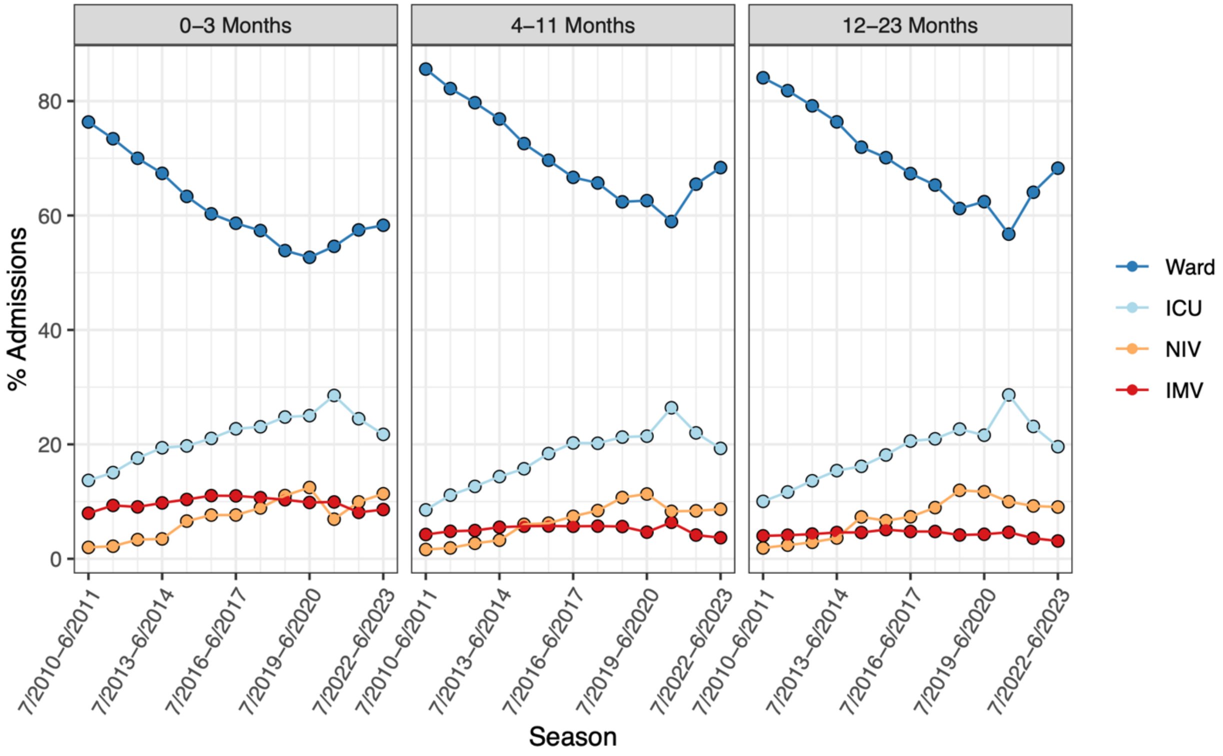

© 2023 Remien KA et al. *JAMA Network Open*.

eFigure 2. Seasonality of Bronchiolitis Admissions Between 2010-2023 With Ensemble Forecast Model Fitting Data

The x-axis shows the month and year. The y-axis shows the number of bronchiolitis admissions. The actual number of admissions is displayed in red. The fitting data for the ensemble forecasting model is shown in the black line. The vertical dashed line shows the model prediction interval. The blue line represents the model prediction, with the dark blue and light blue shaded regions representing the model 80% and 95% confidence intervals, respectively. ARIMA: Autoregressive Integrated Moving Average. NN: Neural Network. MAE: Mean Average Error. RMSE: Root Mean Squared Error.

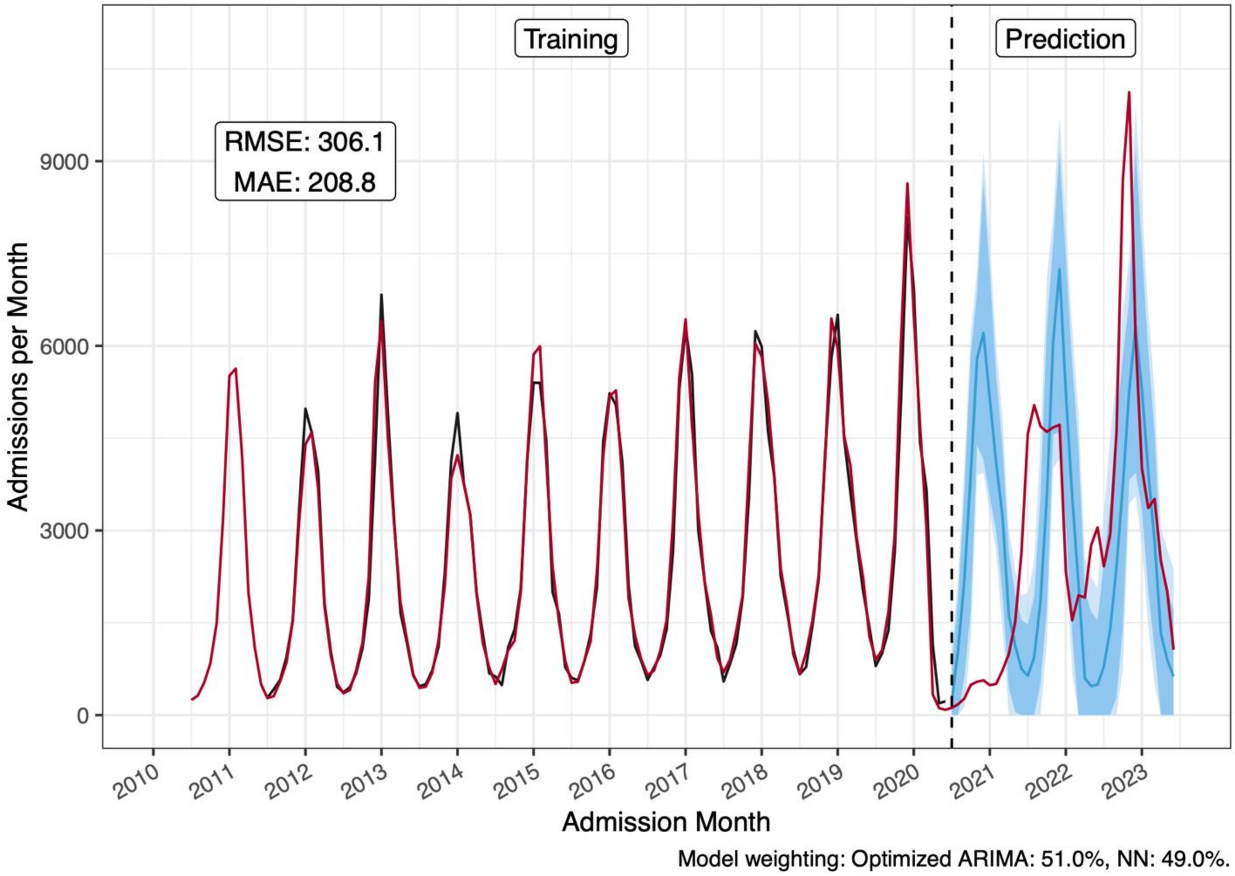

© 2023 Remien KA et al. *JAMA Network Open*.

eFigure 3. Bronchiolitis Hospital Admissions, Bed Utilization, and Cost by Season for Sensitivity Analysis Excluding Complex Chronic Conditions

The x-axis shows the bronchiolitis season. The y-axis shows the number of hospital admissions (upper left), ICU admissions (upper right), hospital days (lower left), and inflation-adjusted cost (lower right). The bar height represents the value for each panel, and the number within the bar shows the value rounded to the nearest whole number. Please note that each facet has a different y-axis scale to show trends.

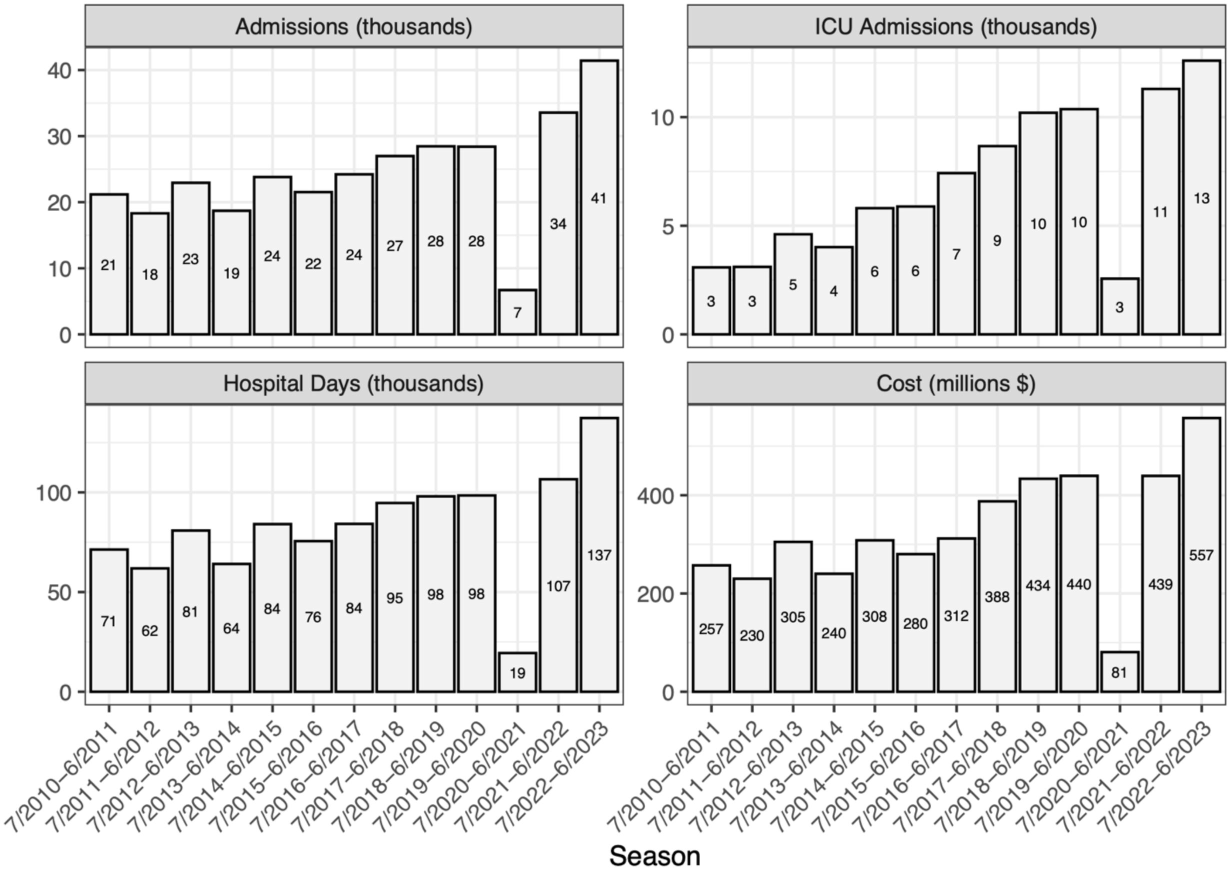

© 2023 Remien KA et al. *JAMA Network Open*.

eFigure 4. Bronchiolitis Hospital Admissions, Bed Utilization, and Cost by Season for Sensitivity Analysis Including Only First Admission per Patient

The x-axis shows the bronchiolitis season. The y-axis shows the number of hospital admissions (upper left), ICU admissions (upper right), hospital days (lower left), and inflation-adjusted cost (lower right). The bar height represents the value for each panel, and the number within the bar shows the value rounded to the nearest whole number. Please note that each facet has a different y-axis scale to show trends.

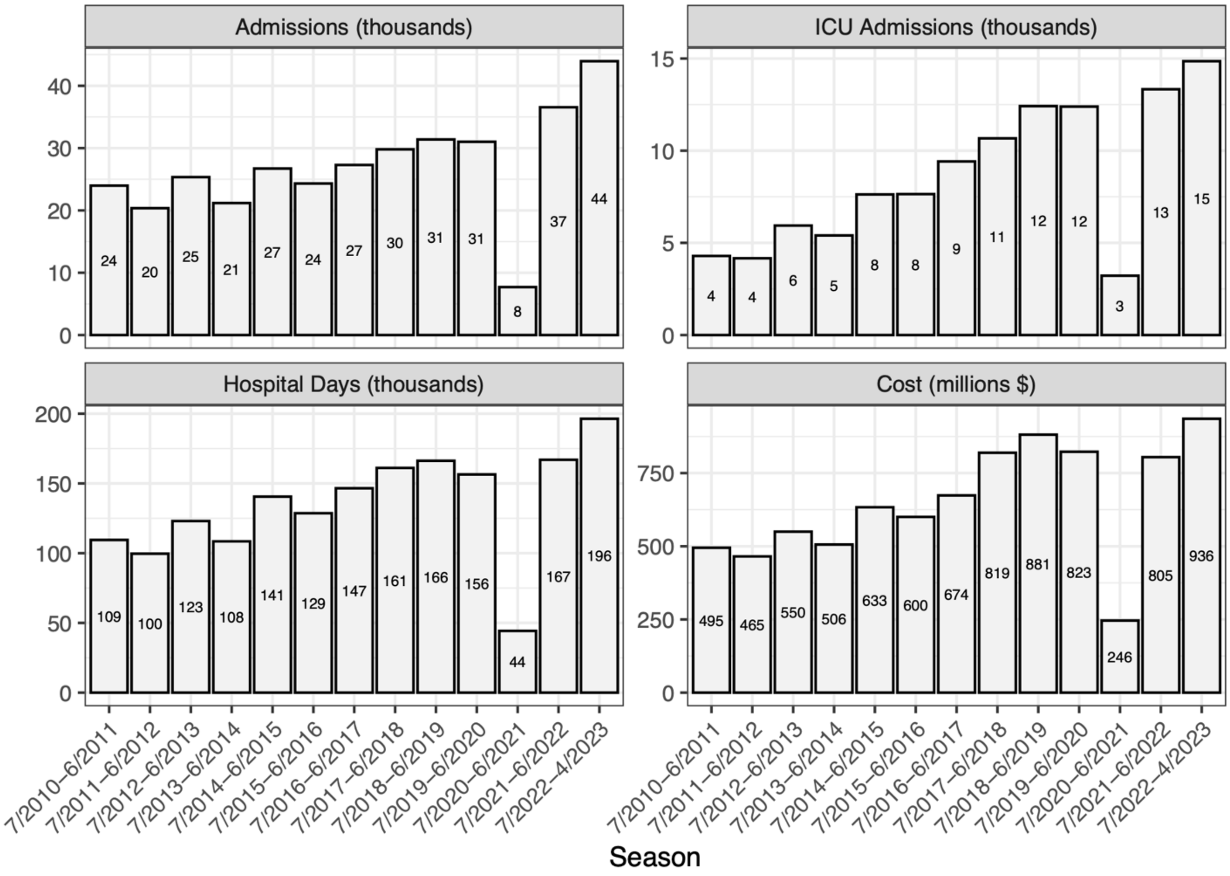

© 2023 Remien KA et al. *JAMA Network Open*.

eFigure 5. Bronchiolitis Hospital Admissions, Bed Utilization, and Cost by Season for Sensitivity Analysis Including Bronchiolitis and Viral Pneumonia for Patients Aged Younger Than 5 Years

The x-axis shows the bronchiolitis season. The y-axis shows the number of hospital admissions (upper left), ICU admissions (upper right), hospital days (lower left), and inflation-adjusted cost (lower right). The bar height represents the value for each panel, and the number within the bar shows the value rounded to the nearest whole number. Please note that each facet has a different y-axis scale to show trends.

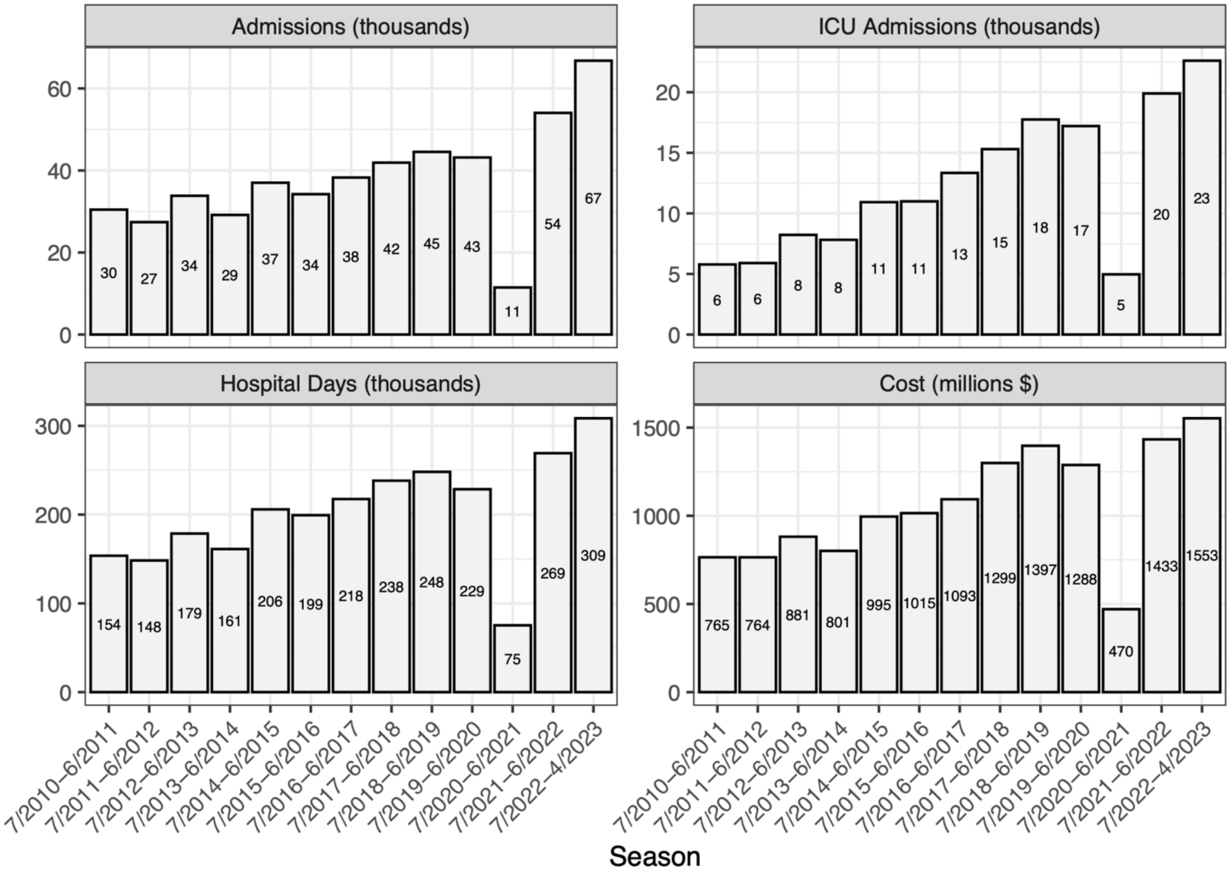

© 2023 Remien KA et al. *JAMA Network Open*.

eFigure 6. Annual Bronchiolitis Admissions by Age Group for Sensitivity Analysis Including Bronchiolitis and Viral Pneumonia for Patients Aged Younger Than 5 Years

The x-axis shows the bronchiolitis seasons. The y-axis shows the number of admissions per season. The colored lines represent each age category. The numbers below the lines represent the number of admissions, rounded to the nearest 100. The colors correspond to the age categories on the line plot. The legend specifies the color for each age group and corresponds both to the lines and to the numbers below the line chart.

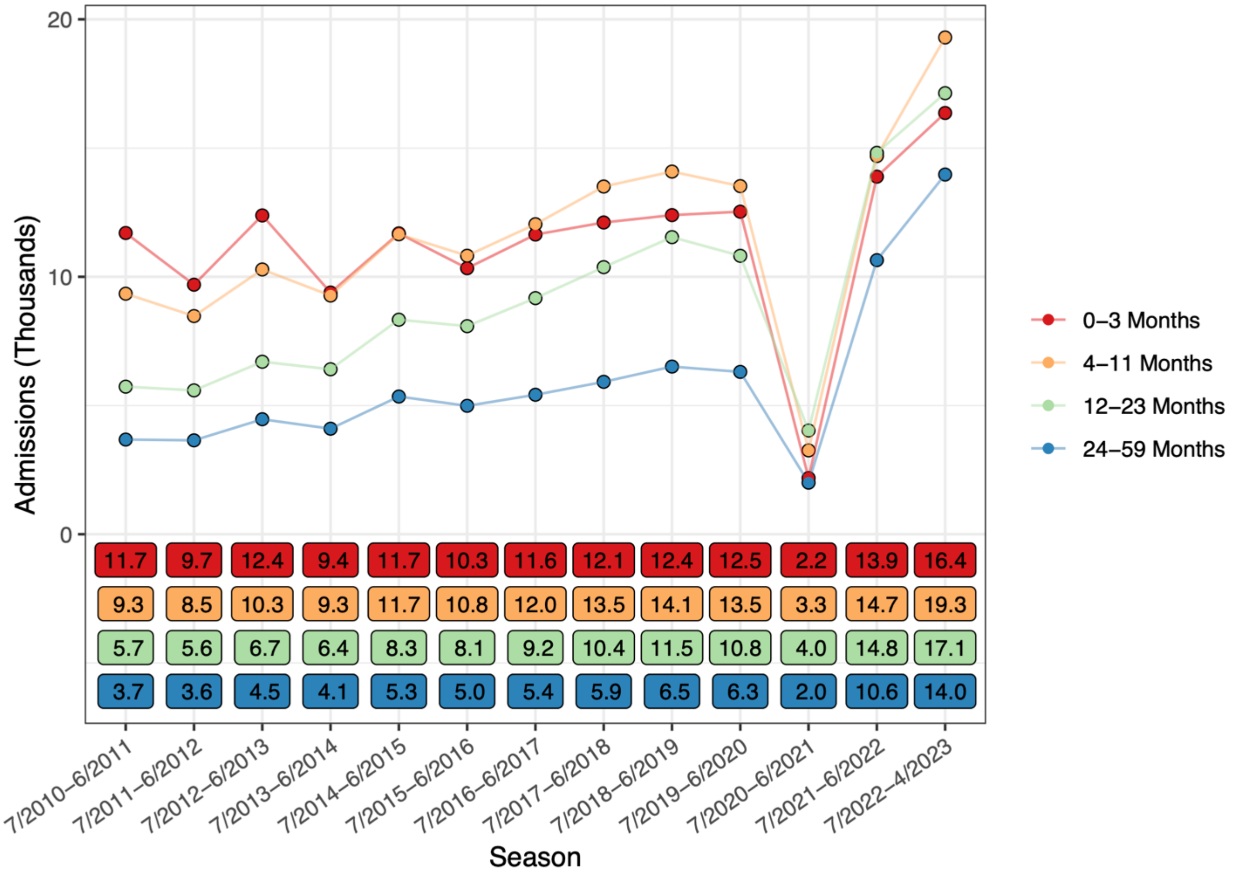

© 2023 Remien KA et al. *JAMA Network Open*.
