# Supplementary material for: Admissions for Bronchiolitis at Children’s Hospitals Before and During the COVID-19 Pandemic
Source: JAMA Netw Open. 2023 Oct 26;6(10):e2339884. doi: 10.1001/jamanetworkopen.2023.39884 (PMC10603547; doi:10.1001/jamanetworkopen.2023.39884)
Supplement: Supplement 2. — Data Sharing Statement [file jamanetwopen-e2339884-s002.pdf]

Data Sharing Statement

Remien. Admissions for Bronchiolitis at Children's Hospitals Before and During the COVID-19 Pandemic. *JAMA Netw Open*. Published October 26, 2023.
doi:10.1001/jamanetworkopen.2023.39884

Data
Data available: No

Additional Information
Explanation for why data not available: The data for this study is owned by the Children's Hospital Association. Any hospital may join at: https://www.childrenshospitals.org/about-cha/about/membership
